# Plasma lipid profile in Nigerians with high - normal blood pressure

**DOI:** 10.1186/1756-0500-7-930

**Published:** 2014-12-18

**Authors:** Hadiza Saidu, Kamilu Musa Karaye, Basil N Okeahialam

**Affiliations:** Department of Medicine, Bayero University/Aminu Kano Teaching Hospital, Kano, Nigeria; Department of Medicine, Jos University Teaching Hospital, Jos, Nigeria

**Keywords:** High-normal blood pressure, Plasma lipids, Nigerians

## Abstract

**Background:**

High blood pressure levels have been associated with elevated atherogenic blood lipid fraction, but epidemiological surveys often give inconsistent results across population sub-groups. To determine the extent to which there are differences in lipid profile based on blood pressure levels, we assessed lipid profile of subjects with high-normal blood pressure and compared with those of hypertensives and optimally normal blood pressure.

**Methods:**

The study was a cross–sectional comparative study conducted at Aminu Kano Teaching Hospital, Kano, Nigeria. Fasting lipid levels were examined among randomly selected patients with optimally normal blood pressure (group 1), high – normal blood pressure (group 2) and those with hypertension (group 3). Optimal blood pressure was defined as systolic blood pressure (SBP) of < 120 mmHg/or diastolic blood pressure (DBP) of < 80 mmHg; and high- normal blood pressure as SBP of 130 – 139 mmHg and/or DBP of 85 – 89 mmHg.

**Results:**

A total of 300 subjects were studied, 100 in each group. The mean age of subjects in group 1 was 27.32 ± 8.20 years and 60% were female, while that of group 2 was 34.04 ± 6.25 years, and 53% were female, and that for group 3 was 52.81 ± 13.3 years and 56% were female. The mean total cholesterol (TC) for subjects in group1 (3.96 ± 0.40 mmol/L) was significantly lower than levels in group2 (4.55 ± 1.01 mmol/L); P = <0.001. Subjects in group 3 (5.20 ± 1.88 mmol/L), however had statistically significant higher mean TC when compared with group 2; (P = 0.03). The difference between the groups for low density lipoprotein cholesterol (LDL-C) and triglycerides (TG) followed the same pattern as that of TC, with statistically significant increasing trend across the blood pressure categories. Levels of high density lipoprotein cholesterol (HDL-C) were however similar across the three groups (group 2 versus group 1; P = 0.49, group 2 versus group 3; P = 0.9). Increased TC (>5.2 mmol/L) was absent in group1, but found among 11% of group2 subjects and 40% of those in group 3 (P-value for trend <0.001). Mean fasting plasma glucose (FPG) was 3.8 ± 0.4 mmol/L, 4.7 ± 1.1 mmol/L, 5.1 ± 1.9 mmol/L and for subjects in groups 1, 2 and 3 respectively (p > 0.05 for groups 2 Vs 1 and p <0.001 for groups 2 Vs 3). The differences in mean body mass index (BMI) between the groups followed a similar trend as that of FPG. Multivariate logistic regression analysis showed that FPG, TG and BMI were the strongest predictors of prehypertension [odds ratio (OR) 10.14, 95% CI (confidence interval) 3.63 – 28.33, P = 0.000; OR 5.75, 95% CI 2.20 – 15.05, P = 0.000; and OR 2.03, 95% CI 1.57 – 2.62, P = 0.000 respectively].

**Conclusion:**

The study has shown a significant increase in plasma TC, LDL-C and TG values as blood pressure levels increased from optimally normal, across high-normal to hypertensive levels. There was a similar trend for FPG and BMI, demonstrating the central role that blood pressure plays in these metabolic disorders in Nigerians. These findings are relevant in terms of both prevention and treatment of cardiovascular morbidities and mortality.

## Background

High – normal blood pressure (BP) levels have been found to be associated more frequently than normal BP with other cardiovascular disease (CVD) risk factors such as dyslipidaemia, dysglycaemia, overweight/obesity, micro-albuminuria and increased circulating levels of various inflammatory markers such as C-reactive protein, and homocysteine [1].

The clustering of these CVD risk factors significantly increases cardiovascular disease, a leading cause of morbidity and mortality [1]. However, dyslipidaemia has been recognized as a strong and independent risk factor for CVD [2].

Plasma cholesterol levels vary significantly in various population groups due to differences in geographical locations, cultural, economical and social conditions, dietary habits and genetic make up [2, 3].

The relationship between high-normal BP and other CVD risk factors including plasma lipids has not been well described in Sub–Sahararan Africans including Nigerians. In the present study therefore, we studied plasma lipid levels in patients with high-normal BP compared with hypertensives and those with optimally normal BP. The results of this study will guide judicious utilization of the scarce resources available for health care and other competing needs given that the assay for plasma lipids is expensive and the subjects with high-normal BP are not “CVD patients”.

## Methods

The study was cross sectional and comparative, carried out in Aminu Kano Teaching Hospital (AKTH), a tertiary healthcare institution in Kano State, North- western Nigeria. The study protocol was approved by the research ethics committee of AKTH, before the commencement of the study. The study conformed to the Declaration of Helsinki on investigations involving human subjects [4] .The study was explained to the patients in English and also in the local language (Hausa) and written consent obtained before enrollment into the study.

### Patient selection

The study population comprised of patients at least 18 years of age, attending the Cardiology and General Outpatient (GOP) clinics of the Hospital. There were 3 patient groups.

Group 1 – Subjects with optimally normal blood pressure who presented to GOP with minor ailments;

Group 2 – Subjects with high - normal blood pressure who also presented to the GOP with minor ailments; and

Group 3 – Hypertensive subjects on treatment and attending the cardiology clinic (first comparative group); and

One hundred patients were selected and evaluated in each group as described below. This sample size was calculated based on the prevalence of high-normal BP of 16.9% in a community-based study in Edo State, Nigeria, using a validated formula [5, 6]. On each of the clinic days, and for each of the groups, numbers were allocated to all patients attending the GOP and a maximum of ten were selected using simple random sampling method by balloting. This was repeated weekly until the desired sample size was attained. Similarly this sampling method was used to select hundred patients being managed for hypertension (Group 3) at the Cardiology clinic during the same period.

Patients already selected previously were excluded if they visited any of the clinics again for another consultation. Also excluded were subjects less than 18 years of age, pregnant women and those on drugs known to increase blood pressure like steroids and contraceptive pills, or to modify the lipids such as statins.

### Data generation

Blood pressure was measured using mercury sphygmomanometer based on standardized techniques [7].

All participants were fasted overnight for 10 – 12 hours after which venous samples were obtained for plasma concentrations of total cholesterol (TC) and fasting plasma glucose (FPG). Samples for total cholesterol were placed in labeled plain universal bottles while those samples for fasting plasma glucose were placed in fluoride oxalate bottles. They were analyzed in the hospital Chemical Pathology laboratory using the auto-analyzer machine (Chiron Diagnostic- Bayer, England). Body mass index (BMI) was calculated from the weight in kilograms taken with minimal clothing using a standardized weight scale and divided by the square height in meters. Height was taken using stadiometer without shoes or head gear.

Optimal blood pressure was defined as systolic blood pressure (SBP) of < 120 mmHg and/or diastolic blood pressure (DBP) of < 80 mmHg; high- normal blood pressure as SBP of 130 – 190 mmHg and/or DBP of 85 – 89 mmHg; while hypertension was defined as SBP of ≥ 140 mmHg and/or DBP of ≥ 90 mmHg, using 2003 World Health Organization/ International Society of Hypertension (WHO/ISH) guidelines [8]. Dyslipidaemia was defined as the presence of any of high total cholesterol (TC) (>5.2 mmol/L), high low- density lipoprotein cholesterol (LDL-C) (>3.38 mmol/L) or low high- density lipoprotein cholesterol (HDL- C) (<1.0 mmol/L in men or if ≤ 1.3 mmol/L in women), and triglycerides (TG) ≥ 1.7 mmol/L, based on the Adult Treatment Panel III (ATP III) guidelines [9]. BMI was considered to be increased when ≥ 25 Kg/m^2^ (overweight; BMI of 25.0 – 29.9 Kg/m^2^ and obesity; BMI ≥ 30 Kg/m^2^) [10].

### Statistical analysis

Data analysis was done using SPSS version 19.0. Quantitative variables were expressed as means and standard deviations. Qualitative variables were expressed as percentages. The chi-square test or Fisher’s exact test where applicable were used in comparing proportions, while Student’s t-test was used to compare means. Logistic regression analysis was used to assess the association of age, anthropometric and serum parameters to blood pressure (high-normal BP or optimal BP). A p-value of ≤0.05 was considered significant.

## Results

There were 100 patients in each of the three groups. The mean age of the subjects in group 1 was 27.32 ± 8.2 years, for group 2, 34.04 ± 6.25 years and 52.18 ± 13.3 years for group 3. The subjects in group 2 were significantly older than those in group 1 (P = < 0.001) while subjects for group 3 were significantly older than those in group 2 (P = < 0.001). There was however no significant statistical differences in gender distribution between the three groups. (P = 0.3) for each comparison. The other baseline characteristics are shown in Table 1.

**Table 1:** **Comparison of the baseline characteristics of the study population**

Variable	Group 1	Group2	Group3	P-value
	Optimal BP	High- normal BP	Hypertensives	
Age (years)	27.32 ± 8.2	34.04 ± 6.25	52.18 ± 13.3	1vs2 (<0.001)*
				2vs3 (<0.001)*
SBP (mmHg)	105.62 ± 7.15	135.25 ± 1.85	143.32 ± 14.97	1vs2 (<0.001)*
				2vs3 (<0.001)*
DBP (mmHg)	70.26 ± 4.58	86.61 ± 0.94	86.76 ± 9.24	1vs2 (<0.001)*
				2vs3(<0.001)*
BMI (Kg/m^2^)	26.55 ± 2.20	22.75 ± 2.20	26.55 ± 4.17	1vs2 (<0.001)*
				2vs3 (0.5)
FPG (mmol/L)	3.76 ± 0.40	4.68 ± 1.09	5.08 ± 1.94	1vs2 (<0.001)*
				2vs3 (0.690

Key: *P-value statistically significant; SBP, systolic blood pressure; DBP, diastolic blood pressure; BMI, body mass index; FBS, fasting plasma glucose; TC, total cholesterol. All values are expressed as Mean ± Standard deviation.

Plasma lipids for the three groups are presented in Table 2. There was a clear gradient of plasma lipids concentrations rising from subjects with optimally normal BP, across those with high-normal BP, to the group with hypertension.

**Figure 1:**
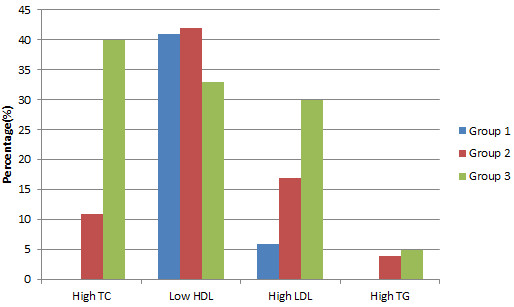
**Pattern of dyslipidaemia among the study and comparative groups.** Key: Blue, Optimal; Red, High- normal; Green, Hypertension. High TC (>5.2 mmol/L) was found in none of subjects with optimal BP, 11% of subjects with high-normal BP and 41% of hypertensives. Low HDL (<1.0 mmol/L in men and < 1.2 mmol/L in women) was found in 41% of subjects with optimal BP, 42% of subjects with high-normal BP and 33% of hypertensives. High LDL (>3.33 mmol/L) was found in 6% of subjects with optimal BP, 17% of subjects with high-normal BP and 30% of hypertensives. High TG (>1.7 mmol/L) was found in none of subjects with optimal BP, 4% of subjects with high-normal BP, 24% of hypertensives.

Comparing the proportion of increased TC, high LDL-C, low HDL-C and high TG, there were statistically significant differences between both groups 1 and 2; and groups 2 and 3 except for HDL-C and TG between groups 2 and 3 (P = 0.24 and 0.6) . The pattern of dyslipidaemia among the three groups is shown in Figure 1.

**Table 2:** **Comparison of means of lipid profiles of subjects with Optimal BP, high – normal BP and hypertension**

Variable	Group 1 (Optimal BP)	Group 2 high-normal BP)	Group 3 (Hypertension)	p-value
TC	3.96 ± 0.40	4.55 ± 1.01	4.68 ± 0.1.09	1 vs2 (<0.001)*
				2 vs3 (0.03)*
HDL-C	1.29 ± 0.54	1.32 ± 0.36	1.36 ± 1.26	1 vs2 (0.9)
				2 vs3 (0.49)
LDL-C	2.07 ± 0.70	2.53 ± 1.12	3.01 ± 1.48	1 vs2 (<0.001)*
				2 vs3 (0.01)*
TG	1.20 ± 0.36	1.50 ± 0.73	1.72 ± 0.83	1 vs2 (<0.001)*
				2 vs3 (0.05)*

Key: *p-value statistically significant; TC; total cholesterol, HDL; high density lipoprotein, LDL; low density lipoprotein, TG; triglycerides. All values are expressed as means ± standard deviations.

The results of multivariable analysis showed that age, BMI, FPG and TG were predictors of high-normal BP (see Table 3).

**Table 3:** **Determinants of high-normal BP Vs Optimal BP from a Multivariable logistic regression model**

Variable	OR	95% CI	P- value
Age	1.16	1.08 – 1.25	0.001
BMI	2.06	1.57 – 2.62	0.000
FPG	10.14	3.63 – 28.33	0.000
TG	5.75	2.20 – 15.05	0.000

Key: OR; odds ratio, CI; confidence interval, BMI; body mass index, FPG; fasting plasma glucose, TG; triglycerides.

## Discussion

The present study assessed plasma lipids among subjects with high-normal BP, and compared the values with those among hypertensive subjects and subjects with optimally normal BP. There was a clear gradient of plasma lipids concentrations rising from subjects with optimally normal BP, across those with high-normal BP, to the group with hypertension. The present study has shown that hypertensives had significantly higher mean total cholesterol (TC) than subjects with high-normal BP, while subjects with high-normal BP had significantly higher mean TC than those with optimal BP. This finding differs from that of Isezuo *et al*. in Sokoto, Northern Nigeria where subjects with pre-hypertension were found to have lower mean TC than the normotensives [11]. It was however similar but lower than what was reported in Singapore [12]. The higher mean TC levels observed in Singapore might be explained by the differences in diet and lifestyle.

Low HDL-C was the most common dyslipidaemia among the groups. Hypertensives had highest mean HDL-C level, followed by subjects with optimally normal BP and then subjects with high-normal BP, a trend similar to what was reported by Lee *et al*. [12]. The findings of higher mean HDL-C among hypertensives when compared with normal controls was documented in earlier studies, in the same region [13, 14]. The generally low HDL-C among the study populations is perhaps the result of low intake of fresh fruits and vegetables by the inhabitants [14]. The same reason might also apply in this study.

The pattern of distribution of mean LDL-C and TG among the groups was however similar to what was reported by previous authors, in which hypertensives had significantly higher mean LDL-C and TG than than subjects with high-normal BP, while subjects with high-normal BP had significantly higher mean LDL-C and TG than those with optimal BP [11, 12, 15].

The present study also shows increases in plasma cholesterol, FPG, and BMI levels as the blood pressure increased, demonstrating the central role that blood pressure plays in these metabolic disorders in Nigerians. Similar to what was obtained by Lee *et al*. in Singapore, the present study showed age, BMI , FBG and TG as significant predictors for high-normal BP [13]. In addition to these, Isezuo *et al*., reported DM, impaired glucose tolerance and cigarette smoking as significant predictors of both pre-hypertension and hypertension [12].

These findings are relevant in terms of both prevention and treatment of cardiovascular morbidities and mortality. In principle, many of the patients we studied could benefit from the polypill. The use of the “polypill” particularly in low income countries like ours might lower cost and increase efficiency and adherence by delivering multiple intervention in a single pill, than same medications prescribed and taken separately. Most polypill contain one to three antihypertensive agents, a statin and some in addition contain aspirin. This idea has still not been widely studied and implemented. There are several challenges to make a successful polypill. Technically, the agents must be stable when combined, and there must be no adverse reactions when the medications are taken together and at same time of the day. Furthermore, it needs to be produced, marketed and distributed, and it must be economically viable.

### Limitations of the study

Although the sample size in the present study was above the estimated minimum, a larger sample size could have demonstrated statistically significant differences between the groups in the BMI and FPG, as was the case in cholesterol levels. Still, the observed trend for the FPG and BMI is relevant in generating hypotheses.

## Conclusions

The present study has shown that dyslipidaemia is common among subjects with high-normal BP. There was a clear gradient of plasma lipids concentrations rising from subjects with optimally normal BP, across those with high-normal BP, to the group with hypertension. There was a similar trend for FPG and BMI.

This study forms a basis for larger population based studies to determine plasma lipid profile among subjects with high- normal BP in this environment. We also recommend that individuals with high-normal BP should be screened for other CVD risk factors and have them corrected through initiation of lifestyle measures in order to prevent them developing hypertension and other complications.

## Abbreviations

AKTH: Aminu Kano Teaching Hospital
BMI: Body mass index
BP: Blood pressure
CVD: Cardiovascular disease
DBP: Diastolic blood pressure
DM: Diabetes mellitus
FPG: Fasting plasma glucose
GOP: General out-patient
HDL –C: High density lipoprotein cholesterol
LDL-C: Low density lipoprotein cholesterol
SBP: Systolic blood pressure
TC: Total cholesterol
TG: Triglycerides
WHO: World Health Organization
WHO/ISH: World Health Organisation / International Society of Hypertension.
